# The Genus *Caesalpinia* L. (Caesalpiniaceae): Phytochemical and Pharmacological Characteristics

**DOI:** 10.3390/molecules17077887

**Published:** 2012-06-29

**Authors:** João L. Baldim Zanin, Bianca A. de Carvalho, Paloma Salles Martineli, Marcelo Henrique dos Santos, João Henrique G. Lago, Patrícia Sartorelli, Cláudio Viegas, Marisi G. Soares

**Affiliations:** 1 Institute of Chemistry, Federal University of Alfenas, 37130-000, Alfenas, MG, Brazil; 2 Institute of Environmental, Chemical and Pharmaceutical Sciences, Federal University of São Paulo, 09972, Diadema, SP, Brazil

**Keywords:** *Caesalpinia*, terpenoids, phenolic derivatives, biological activity

## Abstract

The genus *Caesalpinia* (Caesalpiniaceae) has more than 500 species, many of which have not yet been investigated for potential pharmacological activity. Several classes of chemical compounds, such as flavonoids, diterpenes, and steroids, have been isolated from various species of the genus *Caesalpinia*. It has been reported in the literature that these species exhibit a wide range of pharmacological properties, including antiulcer, anticancer, antidiabetic, anti-inflammatory, antimicrobial, and antirheumatic activities that have proven to be efficacious in ethnomedicinal practices. In this review we present chemical and pharmacological data from recent phytochemical studies on various plants of the genus *Caesalpinia*.

## 1. Introduction

Animal-, mineral-, and plant-derived therapeutic natural products have served as the main source of drugs throughout human civilization [1]. Medicinal substances from plants have been one of the richest sources of organic compounds, contributing significantly to the supply of new chemical entities that have been applied in medicines, cosmetics, foods, and agrochemicals [2,3].

There are several reasons for researching medicinal plants: (1) to gain knowledge about the medicinal potential of native plant diversity; (2) to establish a rational basis for the medicinal use of particular plant species; (3) to develop herbal medicines that are low-cost and exhibit relevant activity; (4) to discover new prototypes for drugs; and (5) to gain information regarding traditional medicines. Medicinal plants are potential sources of bioactive molecules that possess novel structures and mechanisms of action. These innovative features have motivated the pharmaceutical industry to direct research toward the development of herbal medicines [4].

In this context, the minimally studied genus *Caesalpinia* (Caesalpiniaceae) consists of a virtually inexhaustible source of bioactive metabolites within the more than 500 species distributed worldwide. Many of these species are endemic. For example, Brazil-wood (*C. echinata*) exists only in Brazil and played an important role in the history of the country. *C. pulcherrima* is native to Central America, with examples in various other parts of the World, and has been widely used in folk medicine, due to its emmenagogic and abortifacient action. Other species, such as *C. sappan* and *C. bonduc*, have been used for the treatment of inflammation and improving blood circulation, along with serving as an antimalarial, an anthelmintic for the treatment of jaundice and as a digestive [5,6,7].

Phytochemically, several classes of compounds were isolated from plants of genus *Caesalpinia*, mainly flavonoids, diterpenes, and steroids. From a pharmacological point of view, these species have antiulcer, anticancer, antidiabetic, anti-inflammatory, antimicrobial, and antirheumatic activities among others, confirming information derived from ethnopharmacological studies [8]. Due to the diversity of chemical constituents and medicinal importance described in the literature, this work will discuss phytochemical and pharmacological characteristics of the genus *Caesalpinia*. 

## 2. Phytochemicals and Pharmacological Aspects

*Caesalpinia L.* is a genus of plants belonging to the subfamily Caesalpinioideae of the family Caesalpiniaceae and consists of more than 500 species, which are mostly woody species occurring in tropical and subtropical zones. Pharmacologically, species of this genus exhibit analgesic, adaptogenic, antiulcer, anthelmintic, antibacterial, insecticidal, antifungal, anti-inflammatory, antipyretic, antioxidant, antiproliferative, antiviral, immunomodulatory, and immunosuppressive activities. Other species, such as *C. sappan*, *C. ferrea*, and *C. bonducella* show antinociceptive effects. In an abdominal writhing test induced by acetic acid in mice, the stem extracts of *C. sappan* significantly inhibited the number of contortions. The ethanolic extract was the most active, with doses of 200 and 400 mg/kg (v.o.) inhibiting the hyperalgesia by 69.71% and 73.33%, respectively, compared with the control group. Inhibitory activity of the petroleum ether (53.16% and 55.16%) and ethyl acetate (62.47 and 68.84%) fractions followed, whereas aspirin (50 mg/kg) inhibited the contortions by 75.22% [9].

The crude extract from fruits of *C. ferrea*, when subjected to the same pharmacological model discussed above, produced a 51% and 88% reduction of abdominal contractions, respectively, for doses of 10 and 20 mg/kg (v.o.). Comparatively, indomethacin (20 mg/kg) reduced 92% of contractions, while the oil from the seeds of *C. bonducella* (400 mg/kg) showed an inhibition of 48.6%, and standard aspirin inhibited the number of contortions by 66.5% [10,11]. The core of the seed extract of *C. bonducella* also showed analgesic activity in this model, inhibiting the contortions by 65.65% at a dose of 300 mg/kg, whereas aspirin showed 67.59% inhibition at a dose of 100 mg/kg [12]. Other studies with *C. bonducella* revealed that the analgesic activity of the flower extract can be attributed to the presence of flavonoids [13].

The evaluation of the anthelmintic activity of the seeds of *C. crista* justified the traditional use of this species in veterinary medicine. The anthelmintic activity of the hydromethanolic crude extract was tested *in vitro* against *Haemonchus contortus* using mature adults in a motility assay and by monitoring the hatching of eggs. This extract demonstrated anthelmintic effects that were both dose- and time-dependent in causing the mortality of worms and the inhibition of egg hatching (LC_50_ = 0.134 mg/mL). The maximum reduction *in vivo* of eggs per gram (EPG) was recorded as 93.9% for the tested extract of *C. crista* at 3 g/kg, while the standard anthelmintic, levamisole (7.5 mg/kg), showed 95.1 to 95.6% reduction in EPG [14]. Other studies reported in the literature indicated that *C. bonduc* (L) Roxb. and *C. major* extracts exhibited also anthelmintic activity [8,15,16].

A well-known species of the genus, *C. pulcherrima* is a legume found in several countries of Central America, South America, and India. *C. pulcherrima* displayed several medicinal properties to treat ulcers, asthma, fever, skin diseases, and tumors. Several compounds, such as diterpenoids, peltoginoids, flavonoids, chalcones, and homoisoflavonoides, have been isolated from this species [17]. In comparative studies of the antimicrobial activity of the fruits of *C. pulcherrima* (Cp), leaves of *Euphorbia hirta* (Eh) and flowers of *Asystasia gangeticum* (Ag), the activity of *C. pulcherrima* (IC_50_ in mg/mL) was higher than the others, against *Proteus vulgaris*—Cp (0.175), Eh (0.200), Ag (0.273), *Bacillus subtilis*—Cp (0.257), Eh (0.296), Ag (0.320), *Staphylococcus aureus*—Cp (0.166), Eh (0.216), Ag (0.230). *Streptococcus faecalis*—Cp (0.221), Eh (0.241), Ag (0.283), *Candida albicans*—Cp (0.211), Eh (0.275), Ag (0.262), *Aspergillus niger*—Cp (0.215), Eh (0.304) Ag (0.317), *Rhizopus oligosporus*—Cp (0.215), Eh (0.304), Ag (0.317) [18].

**Figure 1:**
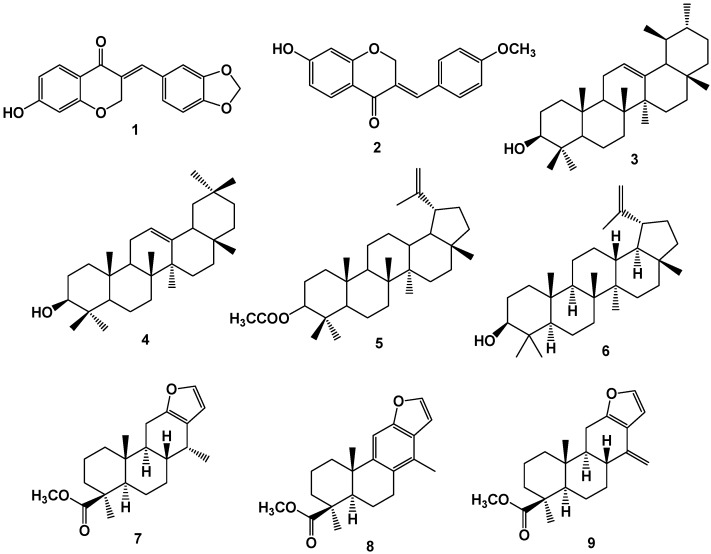
Phenolic compounds and triterpenoids isolated from species of *Caesalpinia* with antibacterial activities.

A homoisoflavonoide isolated from the aerial parts of *C. pulcherrima*, (3*E*)-2,3-dihydro-6,7-dimethoxy-3-[(3-hydroxy-4-methoxyphenyl) methylene]-4*H*-1-benzopyran-4-one (**1**, Figure 1), exhibited higher antibacterial activity against *S. aureus*, *Klebsiella aerogenes*, and *Chromobacterium violaceum* at a concentration of 100 mg/mL than a concentration of 30 mg/mL [17]. Isobonduceline (**2**), isolated from the same plant, showed expressive activity against *B. subtilis* and *Chromobacterium violaceum* [6]. In another study, the methanolic extract of *C. bonducella* seeds was evaluated for their efficacy in inhibiting growth of Gram-positive and Gram-negative bacteria by the diffusion method for zone of inhibition and determination of minimum inhibitory concentration (MIC). The obtained results indicated that this extract exhibited a similar activity of the standard antibacterial kanamycin. After fractionation, the active constituents were identified as the triterpenoids α-amyrin (**3**), β-amyrin (**4**), lupeol acetate (**5**), and lupeol (**6**) [19]. *C. benthamiana* provided cassane diterpenes with antibacterial activity, including deoxicaesaldekarine C (**7**), bentaminine 1 (**8**), and bentaminine 2 (**9**, Figure 1). Among these compounds, compound **8** presented the highest activity (IC_50_ = 47.0 µM) against *S. aureus* and *Micrococcus flavus* [20].

**Figure 2:**
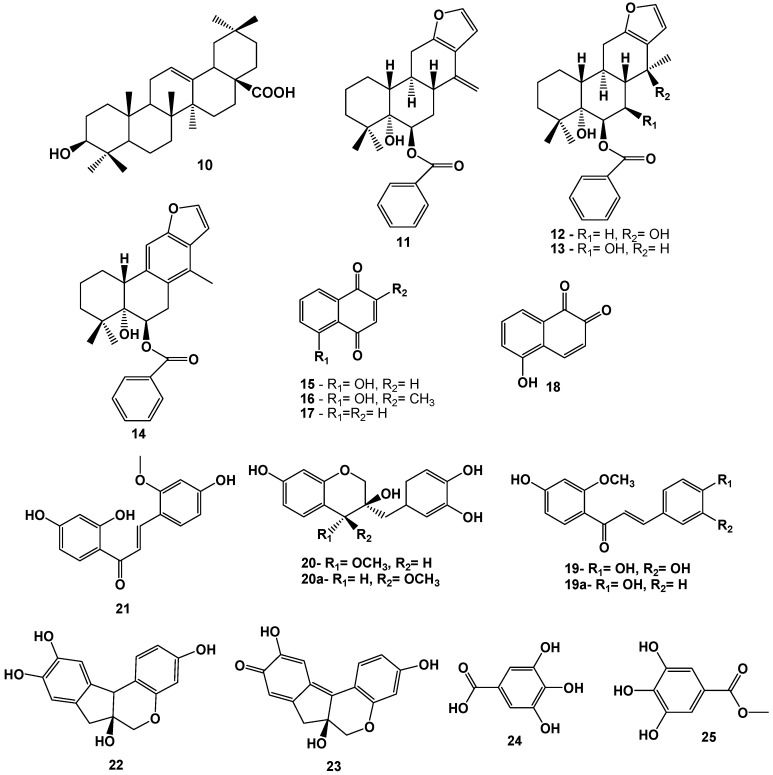
Triterpenoids, naftoquinones and phenolic compounds from species of Caesalpinia showing antimicrobial, antioxidant, antitumoral and citoprotective properties.

*C. paraguaiensis* known as “Guayacán” is another species that showed antibacterial properties. It contains oleanolic acid (**10**) as the active compound against *B. subtilis* and *S. aureus*, with IC_50_ = 8.8 and 64 μg/mL, respectively [21]. Furanic diterpenes isovouacapenol A-D (**11**–**14**, Figure 2) were isolated from *C. pulcherrima* and the antimicrobial activity was evaluated *in vitro* [22], disclosing compounds **11** and **13** as the most active metabolites, exhibiting moderate activity against *S. aureus* and *B. subtilis *at a dose of 30 µg. The leaves of the same species also showed strong antibacterial properties, when tested against several Gram-positive and Gram-negative bacteria [23]. Further studies also indicated that the bark of *C. paraguaiensis* could also elicit antibacterial properties, which were probably related to the presence of ellagic acid and its natural derivatives [24].

The methanol extract of stems of *C. sappan* was tested against five intestinal microorganisms. The constituent with the highest activity against *Clostridium perfringens*, 5-hydroxy-1,4-naphthoquinone (**15**), strongly inhibited growth of the pathogen at doses of 5.0 and 2.0 mg/disc, and moderate growth inhibition was observed at doses of 1.0, 0.5, and 0.25 mg/disc. This compound also showed weak inhibition against the proliferation of *Lactobacillus casei* in 5.0 and 2.0 mg/disc. Activity was found in other similar naphthoquinones, such as 5-hydroxy-2-methyl-1,4-naphthoquinone (**16**), which showed moderate inhibition against *C. perfringens* at 5.0 and 2.0 mg/disc, and 1,4-naphthoquinone (**17**), which significantly inhibited the growth of all bacteria tested at a dose of 5.0 mg/disc. Conversely, 1,2-naphthoquinone (**18**) showed the broadest spectrum of inhibition, being effective against all bacteria tested at 1.0 mg/disc. Prior to these results, Lee and colleagues proposed that substances obtained from the stem of *C. sappan* could be useful as preventive agents against infection by *C. perfringens* [25]. Two neuroprotective compounds were obtained from methanol extract of stems of *C. sappan*, sapanchalcone (**19**) and 4-*O*-methylepisapanol (**20**), together with inactive related compounds methoxychalcone, isoliquiritigenine ether-2-methyl (**19a**), and 4-*O*-methylsapanol (**20a**, Figure 2). At concentrations of 20–40 mM, compound **19** showed cytoprotective effects against oxidative stress induced by glutamate through induction of heme oxygenase (HO)-1 in cells of the hippocampus in rats. Compound **20** also showed moderate neuroprotective activity at 40 mM, while **19** did not exhibited a protective effect against cytotoxicity induced by glutamate in HT22 cells. Pretreatment of HT22 cells with compounds **19** and **20** led to the protection of toxicity mediated by glutamate. Moreover, the use of compound **19** in a non-cytotoxic concentration range of 10–80 µM resulted in concentration- and time-dependent increases of (HO)-1 expression, with compound **19** having more activity than the other metabolites tested [26].

In order to find a natural source for the treatment of oral squamous cell carcinona (OSCC), the potential of the isoliquiritigenine-2'-methyl ether (**21**), which was isolated from the stem of *C. sappan*, was evaluated. Compound **21** showed activity against the cell lines HN4 and HN12, which are involved in OSCC, by dose-dependently inhibiting their growth. The cells treated with **19** exhibited morphological and biochemical changes characteristic of apoptosis [27].

Brazilin (**22**, Figure 2) is a natural pigment commonly used as a dye and found in *C. sappan*, where is particularly abundant (8–22% w/w), as well as in other species of this genus, such as *C. echinata*. Brazil's history is closely linked to this substance, as it is extracted from Brazil-wood and was a source of wealth in the colonial period. Brazilin shows various important biological activities, such as induction of hypoglycemia, anti-platelet aggregation, and induction of immunological tolerance. This compound also induces the expression of (HO)-1 by the activation of antioxidant response elements, which may contribute to cellular defense mechanisms against cell death induced by *tert*-butyl hydroperoxide (*t*-BHP) in House Ear Institute-Organ of Corti 1 (HEO-OC1) cells. This finding suggests that extracts of *C. sappan* and brazilin may be beneficial in the treatment of various diseases associated with oxidative stress [28]. The administration of brazilein (**23**), an oxidation product of brazilin, to rats after cerebral ischemia and the commencement of reperfusion can reduce the area of stroke and improve the neurological score [29,30]. Brazilein also displays cytotoxic activity against human cancer cell lines, such as HepG2 and Hep3B (liver), MDA-MB-231 and MCF-7 (breast), A549 (pulmonary), and CA9-22 (gingival). The literature shows that brazilein also exhibits immunosuppressive activity in lymphocytes from mice, cardiotonic effects in rats, and antioxidant activity [30]. 

*C. ferrea* is a leguminous tree widely distributed in northern and northeastern Brazil, where it is commonly known as “Juca” or “Pau-Ferro”. The aqueous extract of fruits from this specie is used in the treatment of diabetes and coughs and also exhibits antifungal, antiulcerogenic, anti-inflammatory, and analgesic properties. The antitumor effects promoted by the fruit of *C. ferrea* were tested *in vitro* by activation of the Epstein-Barr-Early Antigen Virus (EBV-EA), which causes mononucleosis and other diseases, such as malignancy. The active constituents were identified as gallic acid (**24**) and methyl gallate (**25**, Figure 2). A strong inhibition was reported for **25**, which completely prevented the activation of EBV-EA to a concentration of 22 ng/mL [31]. Antiulcer and toxic effects of stem dry extract of *C. ferrea* were also evaluated. Oral and intraperitoneal administration of 400 mg/Kg of the extract in rats resulted in significant reduction of ulcer index in 50 and 29%, respectively. This effect was pointed as a consequence of inhibition of gastric secretion in the pylorus-ligated rat. Moreover, the negative results on analgesia, sleeping time and spontaneous motor activity tests, were indicative of absence of centrally acting components related to ulcer action. Considering the cicatrizant properties of tannins and that phytochemical studies revealed 4.7% of saponins and 9.2% of tannins present in composition of the stem extract of *C. ferrea*, these classes of metabolites were considered as the probably acting antiulcer components [32,33].

**Figure 3:**
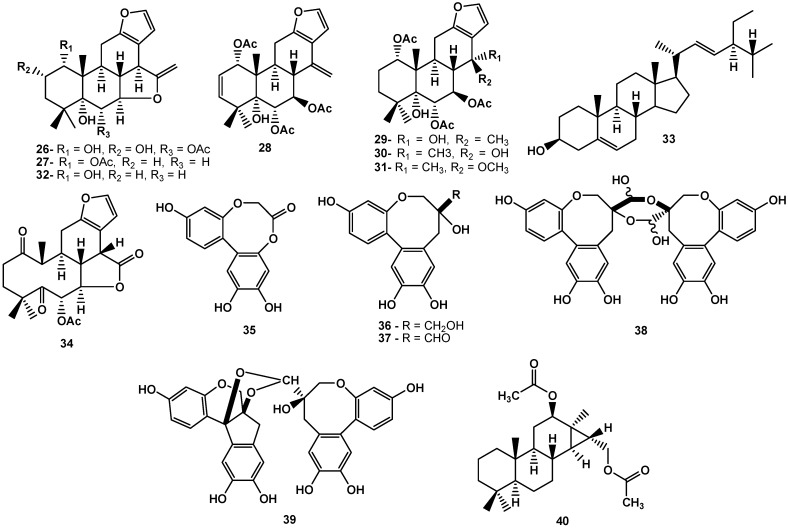
Triterpenoids and protosappanins isolated from species of *Caesalpinia *that exhibited a large spectrum of biological properties, including antiviral and antiinflammatory activities.

Another representative of the genus, *C. minax*, is used in Traditional Chinese Medicine to treat colds, fever and dysentery. The ethanol extract of seeds from *C. minax* showed *in vitro* antiviral activity against Parainfluenza virus type-3 (PIV-3). From *C. minax* were isolated compounds A (**26**) and B (**27**), which were inactive for antiviral activity. Otherwise, the caesalmins C–G (**28**–**32**) and stigmasterol (**33**), displayed activity against the virus PIV-3. In comparison to the values of the standard test (ribavirin – IC_50_ = 2.6 µg/mL), the resulting IC_50_ values for antiproliferative activity to isolated compounds were 8.2 µg/mL (**28**), 9.6 µg/mL (**29**), 10.3 µg/mL (**30**), 7.8 µg/mL (31), 14.8 µg/mL (**32**) and 37.5 µg/mL (**33**, Figure 3) [34]. Another cassane-type furanoditerpenoid isolated from the seeds of *C. minax*, macrocaesalmin (**34**), was evaluated for its antiviral properties against respiratory syncytial virus (RSV), PIV-3, and influenza A. For RSV, compound **34** exhibited an IC_50_ of 24.2 µg/mL, while that of the control ribavirin was 3.4 mg/mL. This compound showed lower activity against PIV-3 (IC_50_ of 51.9 μg/mL), while that of the control was determined as 2.7 µg/mL. On the other hand, the tested compounds did not show activity against influenza A [35]. The species of the *Caesalpinia* genus with anti-inflammatory activity previously reported are as follows: C. *sappan* [7,25,27,28,36,37], *C. ferrea* [10,31,38,39], *C. bonduc* [8], *C. minax* [40], *C. bonducella* [11,12,13], *C. mimosoides* [41], and *C. digyna* [42]. 

Seven compounds isolated from the methanol extract of *C. sappan* inhibit chemical mediators of inflammation of cell line J774.1 using *in vitro* assay. These compounds, namely, brazilin (**22**), sappanchalcone (**19**) and protosappanins A–E (**35**–**39**, Figure 3), were evaluated for their effects on the inhibition of NO and prostaglandin E2 (PGE2) production; the suppression of TNF-α, interleukin-6 (IL-6), and cyclooxygenase-2 (COX-2); and the expression of nitric oxide synthase (iNOS) induced by mRNA. Compound **22** inhibited NO production without affecting PGE2, unlike **19**, **38**, and **39**, which inhibit both (iNOS and PGE2) production and suppression of TNF-α, IL-6 and COX-2 [7].

Several analogues of brazilein, specifically homoisoflavonoides, were synthesized and evaluated for cytotoxic activity in human cancer cell lines, showing results better than the original molecule. However, compared to Taxol^®^, all analogues were less efficient [30]. 

In studies of bioprospection, natural substances that exhibit inhibitory properties of the biosynthesis of prostaglandins and NO production have been considered to be potential anti-inflammatory and cancer chemopreventive agents. In this context, the extract of *C. sappan* showed more than 70% of inhibition of iNOS and COX-2 [35]. In another study, oral administration (300 mg/kg) of the aqueous extract of fruits from *C. ferrea* showed significant activity in the assay of paw edema in rats induced by carrageenan [10,37]. The crude extract of the seeds of *C. ferrea* did not show acute toxicity in mice, even when administered at the maximum dose (0.3 mL/10 g body weight). Otherwise, this extract showed cellulase and larvicidal activity against *Aedes aegypti* with 85% mortality in less than 24 h, suggesting that this extract can be used as an alternative to fight the mosquito that transmits dengue [43].

The oil from the seeds of *C. bonducella* exhibited anti-inflammatory activity in rats at a dose of 400 mg/kg in a paw edema test induced by carrageenan. The obtained results indicated that after three and four hours of the start of the experiment, the potential was equivalent to that observed of the positive control phenylbutazone at a dose of 100 mg/kg [11]. The bioguided phytochemical study on the extract of the roots of *C. mimosoides* led to the isolation of several diterpene compounds. The anti-inflammatory activity of all compounds was evaluated for inhibitory activity of nitric oxide (NO) induced by LPS (lipopolysaccharide) in the cell line RAW264.7. These results showed that mimosol D (**40**, Figure 3), exhibited a potent inhibitory activity for both inflammatory mediators with IC_50_ values of 3 µM and 6.5 µM for inhibiting the production of NO and TNF-α, respectively [40].

**Figure 4:**
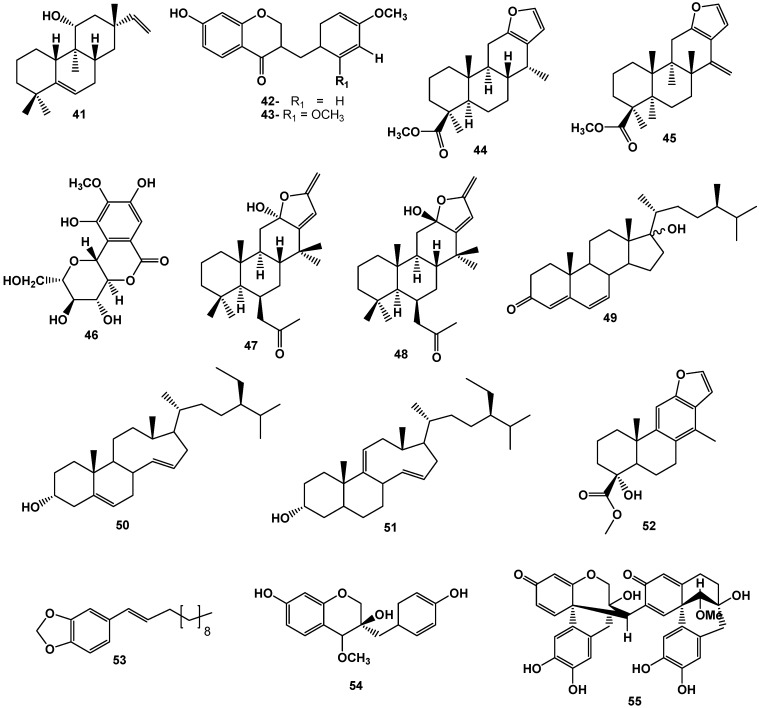
Some triterpenoids and phenolic compounds from species of *Caesalpinia* with antioxidant and enzyme inhibitory activities.

Several species of the genus *Caesalpinia* presented also antimalarial activity. In a series of metabolites isolated from the roots and stems of *C. crista*, *ent*-11b-hydroxy-5,15-rosadiene (**41**, Figure 4) showed antimalarial activity [44]. Similarly, different fractions derived from the extract of the leaves of *C. volkensii* (decoction, maceration in EtOH, petroleum ether, MeOH and H_2_O) were tested for antimalarial activity in comparison to *Ajuga remota*, which is known for its use in traditional medicine. The results showed that *A. remota* is more effective than most fractions of *C. volkensii*. The fraction of decoction was notably active against the chloroquine-sensitive *P. falciparum* (FCA: 20GHA), as was the petroleum ether fraction, which showed activity equivalent to *A. remota* into another species of chloroquine-resistant *P. falciparum* (W2) [45].

Some species of the genus *Caesalpinia*, including *C. bonduc* [8,46,47,48,49], *C. bonducella* [19], and *C. decapetala* [50,51] were also evaluated for antipyretic activity. The extract from seeds of *C. bonducella*, at a dose of 30 mg/kg, significantly reduced pyrexia after 3, 5, and 6 h. The same extract, at a dose of 100 mg/kg, reduced pyrexia in 14.81% within 3 h, 32.10% after 5 h, and 64.2% after 6 h. At a dose of 300 mg/kg this extract was able to reduce the pyretic effect in 10.83%, 18.33% and 33.33%, within 3, 5, and 6 h, respectively. However, it was found to be less potent than the same dose of acetylsalicylic acid used as standard, which reduced the pyrexia in all periods of observation of the assay [12]. The extract from flowers of *C. bonducella* was administered orally at doses of 30, 100, and 300 mg/kg, thereby reducing the pyrexia in adult mice [13]. In another study, it was shown that the oil from the seeds of *C. bonducella* exhibited antipyretic activity compared with paracetamol in a test on pyrexia induced by yeast in rats. At a concentration of 400 mg/kg, the extract was equivalent to the positive control, also showing a significant effect at doses of 200 and 300 mg/kg [11].

In trials to evaluate the potential antioxidant capacity, the ethanolic extract of seeds of *C. bonducella* showed a high free radical-scavenging activity by 2,2-diphenyl-1-picrylhydrazyl (DPPH) with an IC_50_ = 74.73 µg/mL compared to the ascorbic acid (AA, IC_50_ = 26.68 µg/mL). In the activity test of the hydroxyl radical (ASROH), the extract was active with IC_50_ = 109.85 µg/mL, which again was less potent than AA (IC_50_ = 70.79 µg/mL). Similar effects were also observed in the activity assay of radical scavenging NO (ASRON) with IC_50_ values of 102.65 and 65.98 µg/mL for the extract and AA, respectively. In the case of the superoxide radical (ASRS), the extract exhibited an IC_50_ of 89.84 µg/mL while an IC_50 _of 36.38 µg/mL was determined to AA was [15]. Despite these results, additional studies were not performed on fractionation in order to identifying the active constituents.

Conversely, dihydrobonducellin (**42**) and 2-methoxydihydrobonducellin (**43**, Figure 4), isolated from *C. pulcherrima*, showed superoxide radical scavenging activity inhibition values that were better than the antioxidants commonly used as positive controls, with IC_50_ values of 352 µM and 325 µM, respectively, compared with vitamin C (IC_50_ = 852 µM), vitamin A (IC_50_ = 726 µM), BHA (IC_50_ = 966 µM), and BHT (IC_50_ = 381 µM) [52]. Compounds **42** and **43** are approximately three times more potent than the control vitamin C. *In vitro* antioxidant activity of ethanolic extract of the heartwood (CSE) from *C. sappan* and the isolated constituents protosappanin A (**35**), protosappanin B (**36**), and brazilein (**23**), were tested using MDA (malondialdehyde) assay. While vitamin C at a concentration of 0.368 µg/mL inhibited 14.02% of the radical, the CSE showed the same value at a concentration of 0.0006 µg/mL, indicating that CSE is 600-fold more potent than positive control. In this study, it was observed that none of the tested substances was more potent than the CSE because the highest concentration in each case for compounds **35**, **36** and **23** showed 20.3% (0.147 µg/mL) 19.5% (0.102 µg/mL), and 0.54% (0.074 µg/mL), respectively [36], suggesting a synergistic effect.

Extract from the leaves of *C. crista* wss assayed using different methods of analysis of antioxidant activity, showed the following results of IC_50_ in µg/mL: 0.4 ± 0.1 ASROH—control mannitol 0.85 ± 0.02; ASRS 24.9 ± 0.9—control quercetin 47.4 ± 0.7, 33.7 ± 0.8 ASRON—97 ± 3 control curcumin, singlet oxygen-scavenging activity (ASOS) 61 ± 3—control lipoic acid 49 ± 8; hypochlorous acid-scavenging activity 170 ± 4—control AA 198 ± 11; iron ion-chelating activity (IICA) 280 ± 5—control EDTA 1.27 ± 0.05 [53].

Chemical studies in order to identify metabolites with antioxidant activity in *C. benthamiana* revealed the occurrence of diterpenes deoxycaesaldekarin C (**44**) and benthaminin 2 (**45**, Figure 4). The most active compound was **45**, displaying an IC_50_ of 42.7 µM in the assay using DPPH, while an IC_50_ of 74.2 µM was determined to control thiobarbituric acid (TBA) [20,54].

The antioxidant activity of extracts of *C. digyna* and an isolated component, bergenin (**46**), justify the extracts’ in traditional medicine. The extract of the roots of *C. digyna* was demonstrated to be a potent *in vitro* scavenger of free radicals in different models and showed a dose-dependent antioxidant activity, inhibiting lipid peroxidation and increasing such antioxidant enzymes as superoxide dismutase and catalase in the CCl_4_ intoxication model in rats [41]. 

Antidiabetic properties were reported for several species such as *C. sappan* and *C. bonducella*, thereby demonstrating their potential as a drug against *diabetes mellitus*. The extract of the seeds of *C. bonducella* administered at 300 mg/kg in rats showed a significant hyperglycemic action in tests to induce hyperglycemia [55]. This data corroborates the earlier study [56], which demonstrated the ability of the aqueous extract of *C. bonducella* to reduce levels of sugar in the blood between 3 and 5 h after administration of a dose of 250 mg/kg compared to alloxan and streptozotocin used to induce hyperglycemia in rats. 

The extract from seeds of *C. bonduc* was tested for its adaptogenic activity using the models of cold stress and forced swimming in rats and exhibited significant anti-stress activity when administered orally at a dose of 300 mg/kg. The extract also showed effectiveness in controlling the hyperlipidemic condition caused by stress [57]. In addition, *C. bonduc* was evaluated for antiproliferative properties using a model of epidermal hyper-proliferation in psoriasis. To assess the cytotoxic effect of the plant, human keratinocyte HaCaT cells with rapid proliferation were used. With the exception of the decoction, which showed IC_50_ greater than 500 mg/mL, all other extracts showed appreciable antiproliferative activity. The hydroalcoholic extract at 21% was the most potent with an IC_50_ = 77 ± 13 μg/mL, followed by the hydroalcoholic extract at 1% (IC_50_ = 102 ± 10 μg/mL), hydroalcoholic extract at 14% (IC_50_ = 133 ± 11 μg/mL), and hydroalcoholic extract at 80% (IC_50_ = 150 ± 19 μg/mL), in comparison to asiaticoside (IC_50_ = 20.13 mg/mL) used as a positive standard [58]. In addition to activity against psoriasis, other compounds isolated from *C. bonduc*, the epimers caesalpinolida (**47**) and B (**48**), showed antiproliferative activity in breast cancer (MCF-7 and MDA-MB-231), endometrial (Ishikawa), and cervical (Hela) cell lines. Compounds **47** and **48** showed significant inhibition of line MCF-7 with IC_50_ of12.8 µM and 6.1 µM, respectively [59,60]. The *in vitro* evaluation of activity against filaria of the extract of leaves and oils from seeds of *C. bonduc* was conducted by using the microfilariae of *Wuchereria bancrofti* and *Setaria digitata*. Both extracts showed a significant dose-dependent anti-filaric effect at doses of 30, 53 and 80 μg/mL against *W. bancrofti* and 25, 50, and 100 μg/mL for *S. digitata*, while the oil was effective at doses of 47 and 67 μg/mL for *W. bancrofti* and 50 and 100 μg/mL against *S. digitata* [61]. 

The aqueous extract of the stem bark of *C. ferrea* induced hypotension associated with tachycardia in normotensive rats, whereas at a dose of 40 mg/kg, the extract induced transient bradyarrhythmias. Moreover, the aqueous extract from stem bark of *C. ferrea* also induced vasodilation in the mesenteric arteries of rats, which appears to be mediated primarily by potassium channels sensitive to ATP, thereby indicating a possible hypotensive property associated with this ion channel [39]. 

Bioguided chemical study by inhibition assay of glutathione S-transferase (GST) of the fractions of the ethanol extract from barks of *C. bonducela* resulted in the isolation of a new steroid: 17-hydroxycampesta-4,6-dien-3-one (**49**), and four other known compounds: 13,14-*seco*-stigmasta-5,14-dien-3α-ol (**50**), 13,14-*seco*-stigmasta-9(11),14-dien-3α-ol (**51**), caesaldekarin (**52**), and pipatalin (**53**, Figure 4), all of which exhibiting inhibition of GST. The values IC_50_ of these compounds and their derivatives were approximately 57–380 µM and comparable to the inhibition effects of sodium taurocholate, which is an isoprene-derived GST inhibitor (IC_50_ = 398 µM) [8].

*C. benthamiana* is an African tropical plant which aqueous decoction from roots has been used in traditional medicine for many purposes, especially for treatment of erectile dysfunction. Chemical studies have shown that roots of this plant are rich in phenolic compounds, mainly gallic acid and resveratrol, as well as tannins. Results of pharmacological evaluations have shown that the aqueous extract of *C. benthamiana* exhibited significant vasorelaxant activity. In addition, the extract showed also radical scavenging activity against ROS species in cell-free systems and stimulated the NOS expression of mRNA in cellular environments. The levels of polyphenols and total tannins from *C. benthamiana* were determined colorimetricaly to be 15.5% and 9.9%, respectively [62].

Other compounds, such as sappanchalcone **19** and 3'-deoxy-4-*O*-methylepisapanol (**54**), which were obtained from the methanol extract from barks of *C. sappan*, were evaluated in cortical cells of rats injured with glutamate at doses of 0.1 to 10 µM. Only compound **54** showed significant activity, attenuating the toxicity induced by glutamate with cellular viability of 50–70% [63,64]. Other compound isolated from *C. sappan*, neo-sappanone (**55**, Figure 4), was evaluated for inhibition of xanthine oxidase and showed a dose-dependent inhibitory activity with IC_50_ of 29.7 µM, 10-fold less potent than the positive control allopurinol (IC_50_ = 2.6 µM). This finding suggests that structural changes of semi-synthesis may potentiate the efficiency of the natural product, enabling the development of an alternative for the treatment of hyperuricemia [65].

## 3. Conclusions

Numerous studies conducted with species of the genus *Caesalpinia* have corroborated their effectiveness as a natural source of new chemical entities and new therapeutical applications, revealed their structural diversity, showen useful properties for the development of traditional medicines, and produced scientific guidance for the use of herbal medicines. However, with approximately 500 species of this genus occurring worldwide, less than 30 of these have been already studied with respect to their phytochemicals and pharmacological activities. Among the metabolites described, including the predominant phenolic derivatives, steroids, triterpenoids, and especially the cassane diterpenes, many of these exhibited antiulcer, anticancer, antidiabetic, anti-inflammatory, antirheumatic, antimicrobial, antibacterial, and cytotoxic activities. Therefore, the results available in the literature to date, when associated with the diversity of metabolites, clearly indicate that chemical/pharmacological research of species belonging to the genus *Caesalpinia* could afford new drug prototypes. The species of *Caesalpinia* that did not have yet been studied may bring valuable benefits to the relentless search for bioactive molecules, which have medicinal action and therapeutic feasibility, thereby allowing the discovery and development of more efficacious drugs that are safer and more affordable.

The contribution of natural products could be even greater if we consider the Brazilian biodiversity, the occurrence of several neglected endemic diseases in several regions of Brazil, and the techniques already being employed in research laboratories at public universities and national research centers. These factors, coupled with government interest in public health, should encourage the search for alternative treatment, control, and eradication of such diseases as dengue, malaria, leishmaniasis, Chagas disease, and others that still have no cure and, or limited therapeutic treatments, besides the contribution of this research to other fields of science, technology and innovation.
